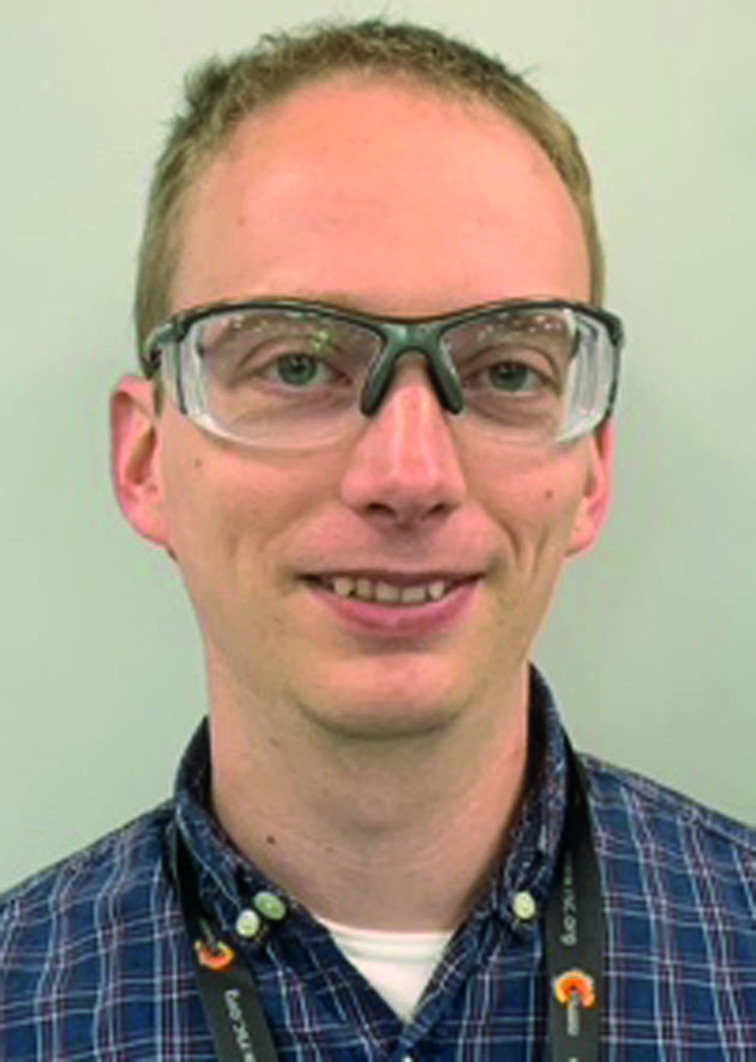# Exploring Supramolecular Frustrated Lewis Pairs

**DOI:** 10.1002/cplu.202400725

**Published:** 2025-02-11

**Authors:** Paige R. Hawkins, Chris S. Hawes, Peter D. Matthews

**Affiliations:** ^1^ School of Chemical and Physical Sciences Keele University Keele ST5 5GB

**Keywords:** Supramolecular, Frustrated Lewis pairs, Catalysis, MOFs, COFs

## Abstract

Frustrated Lewis pairs (FLPs) have rapidly become one of the key metal‐free catalysts for a variety of chemical transformations. Embedding these catalysts within a supramolecular assembly can offer improvements to factors such as recyclability and selectivity. In this review we discuss advances in this area, covering key supramolecular assemblies such as metal organic frameworks (MOFs), covalent organic frameworks (COFs), polymers and macrocycles.

## Graphical abstract

Frustrated Lewis pairs (FLPs) are a key metal‐free catalysts for a variety of chemical transformations. Embedding these catalysts within a supramolecular assembly can offer improvements to factors such as recyclability and selectivity. In this review we discuss advances in this area, covering key supramolecular assemblies such as metal organic frameworks (MOFs), covalent organic frameworks (COFs), polymers and macrocycles.

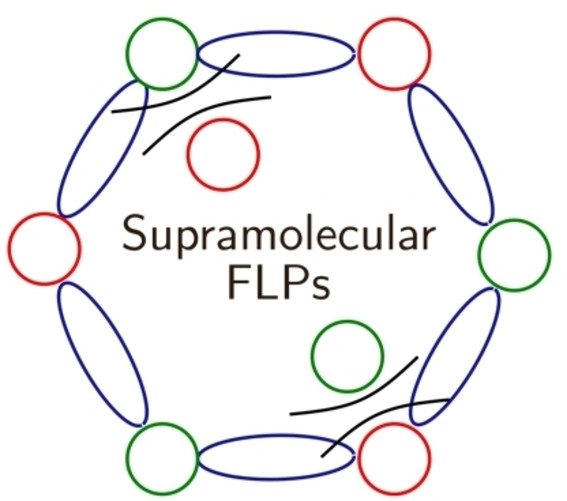

## 1 Introduction

Frustrated Lewis pairs (FLPs) are mixtures of Lewis acids and Lewis bases that have been prevented from forming a classical Lewis adduct. This might be in intermolecular pairs, where steric bulk often plays an important role, or through holding the Lewis acid and base far apart intramolecularly. The concept of FLPs was pioneered by Stephan and co‐workers in the mid‐2000s with their initial report of an intramolecular boron/phosphorus spatially separated pair that activated dihydrogen, which was followed by a demonstration that ^t^Bu_3_P and B(C_6_F_5_)_3_ (BCF) could activate H_2_ or add to olefins.[^1^, ^2^, ^3^] Since those three seminal works, molecular FLPs have been extensively studied.

In the 18 years since the first activation of H_2_ by a FLP, the scope of FLP reactivity has been expanded to encompass a vast variety of unsaturated substrate reductions, and activation of CO, CO_2_, N_2_O, SO_2_, olefins, alkynes, and C−H bonds.[^4^, ^5^, ^6^, ^7^, ^8^, ^9^, ^10^, ^11^, ^12^, ^13^, ^14^] A number of high‐quality reviews cover these topics and we direct the reader's attention to these.

**Figure 1:**
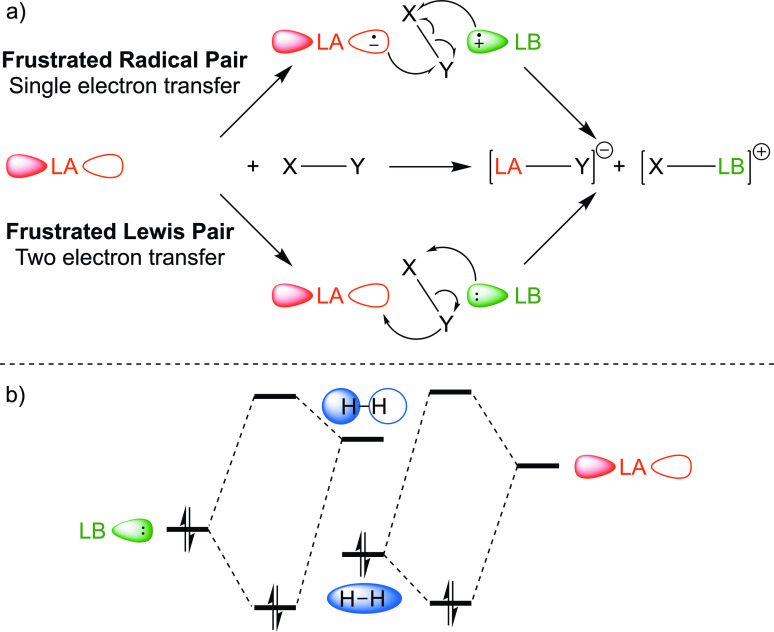
a) Schematic overview of FLP mediated activation of a X−Y bond, which can occur homolytically (top) *via* a single electron transfer process, or heterolytically (bottom) through a two electron transfer process. b) Synergistic interaction of the Lewis base LUMO and Lewis acid HOMO with the H−H σ* and H−H σ orbitals respectively.

The archetypal reactivity of FLPs is the cleavage of a bond either heterolytically (frustrated Lewis pair, two electron transfer) or homolytically (frustrated radical pair, single electron transfer) (Figure 1a).^[14]^ For H_2_, this is brought about by an interaction between the HOMO of the Lewis base and the H−H σ*, and the LUMO of the Lewis acid and the H−H σ (Figure 1b). H_2_ must be polarised prior to the synergistic orbital interactions, and electrostatic interactions between the Lewis acid/base must be considered.^[11]^

The immobilisation of molecular catalysts generally offers an improvement in stability, recyclability and therefore net cost compared to their homogeneous congeners.[^15^, ^16^, ^17^, ^18^, ^19^, ^20^, ^21^, ^22^, ^23^] A secondary, but no less important, benefit is the geometric constraint that such immobilisation can confer on the active catalyst site. This constraint can start to have a substantial impact on the performance of the active catalyst through increasing the performance of changing the chemo‐/regio‐/enantioselectivity of the reaction.[^24^, ^25^, ^26^, ^27^, ^28^, ^29^, ^30^, ^31^, ^32^, ^33^]

This is particularly important in FLP catalysts, where the intrinsic activity of the catalytic pair is governed by the spatial separation between the Lewis acid and base.[^5^, ^6^, ^10^, ^34^, ^35^, ^36^]

Anchoring FLPs in supramolecular assemblies is a new and growing area of interest, with a full gamut of strategies ranging from metal–organic frameworks (MOFs)^[37]^ and zeolites^[38]^ through to polymers,^[39]^ macrocycles^[40]^ and cages. The structural and chemical diversity on show has led to some exciting and innovative results and in this review we will highlight the key areas that are under development and the areas that we consider ripe for future exploitation.

## 2 Porous Networks

### 2.1 Metal–Organic Frameworks

Metal–organic frameworks (MOFs) are porous crystalline materials that are renowned for their structural diversity, tunability, and selectivity in various chemical reactions.[^42^, ^43^, ^44^, ^45^, ^46^, ^47^, ^48^, ^49^, ^50^, ^51^, ^52^] MOFs are well established as catalyst supports for organic transformations, utilising their intrinsically uniform structures as support platforms for supported‐but‐homogeneous‐in‐function catalysts – thereby combining the advantages of heterogeneous and homogeneous catalysts in one package.[^53^, ^54^, ^55^, ^56^, ^57^] However, the integration of FLPs within these frameworks is a much less studied, though growing area.

Heterogeneous MOF/FLP systems have rapidly gained attention over the last 10 years, with particular focus on catalytic reduction reactions and small molecule activation.[^58^, ^59^, ^60^] In a similar manner to traditional MOF catalysts, FLP‐MOFs are exploited in two different fashions: *i)* post‐synthetic grafting of Lewis acid/base onto the MOF or *ii)* the metal node or organic linker forms at least one part of the Lewis pair. These two different design strategies have resulted in a variety of different catalysts that make the most of the structural advantages of MOFs in different ways.

Post‐synthetic modification of a known MOF is a well‐established enabling strategy in the field for broadening the functionality of MOFs. Incorporating a diverse range of functional groups, and in particular phosphines/boranes which are commonly used in FLP chemistry, can be a challenge due to the relatively harsh solvothermal conditions of a MOF synthesis. It is substantially easier to either coordinate a new ligand to a vacant metal site, or covalently modify the organic linker in the MOF.[^61^, ^62^]

**Figure 2:**
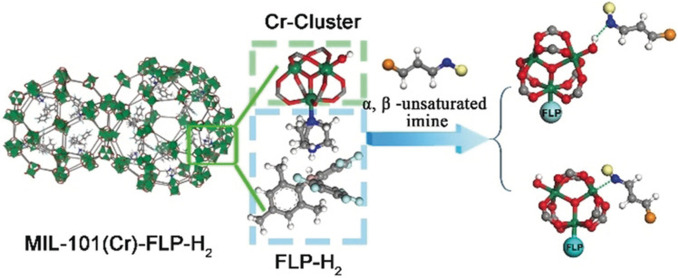
Addition of DABCO to a coordinatively unsaturated Cr centre in MIL‐101(Cr) to form a MOF/FLP with BCF. Copyright 2019 Wiley. Reproduced from Ref. [41] with permission from Wiley.

Post‐synthetic modification to add a Lewis acid/base requires a MOF with a readily accessible metal site or an organic linker with a pendant functional group that points into the pore. The chromium terephthalate based MOF, MIL‐101(Cr)[^63^, ^64^, ^65^] consists of Cr(III) clusters connected by 1,4‐benzenedicarboxylates [molecular formula=Cr_3_(OH)O(BDC)_3_, BDC=1,4‐benzenedicarboxylate] and has a highly porous 3D structure with pores of 29 and 34 Å. The metal clusters are relatively accessible and upon dehydration Cr(III) sites can be exposed. This, in combination with the large pore sizes, have been exploited to allow for the grafting of Lewis bases to the metal, whilst retaining room for a Lewis acid and molecular reactants. Thus, an archetypal design strategy for a FLP‐MOF was demonstrated by Ma's group using MIL‐101(Cr) and 1,4‐diazabicyclo[2.2.2]octane (DABCO) as the Lewis base in conjunction with BCF to promote the hydroborylation of imines with HBpin (Figure 2).^[41]^ Switching the Lewis acid to B(C_6_F_5_)_2_(Mes) allowed the MIL‐101(Cr)/DABCO system to selectively hydrogenate the imine group in α,β‐unsaturated imines under moderate (10 bar) H_2_ pressure.^[41]^ This is the reverse of homogeneous FLPs, which are known to hydrogenate α,β‐unsaturated compounds, but reduction is typically favoured at the C=C bond.[^66^, ^67^] However, this catalyst demonstrated poor performance in the hydrogenation of nitrogen heterocycles, partly due to the lower acidity of B(C_6_F_5_)_2_(Mes).

Changing the chromium coordinated Lewis base to N,N′‐dimethylethylenediamine (DMEDA) affords a MIL‐101(Cr)‐DMEDA system. The addition of BCF gave an effective transfer hydrogenation catalyst for the reduction of ethyl benzylidenemalonate to diethyl benzylmalonate that outperformed the non‐MOF anchored FLP.^[68]^

The rigid and well‐defined nature of MOF pores suggests that one of the advantages of MOF/FLP systems is the ability to exert regio‐ or chemoselectivity. Using a chiral Lewis base is a relatively facile way of controlling the stereoselectivity of a reduction, and the introduction of (*R*)‐2,5‐dihydro‐3,6‐dimethoxy‐2‐isopropylpyrazine with BCF has led to asymmetric hydrogenation of imines^[69]^ and selective enantioselective reduction of α,β‐unsaturated imines.^[70]^

A different class of MOF that has readily functionalisable sites is NU‐1000 [molecular formula Zr_6_(μ_3_‐OH)_8_(−OH)_8_(TBAPy)_2_, TBAPy=1,3,6,8‐tetrakis(*p*‐carboxyphenyl)pyrene]. The metal node [Zr_6_(μ_3_‐OH)_8_(OH)_8_]^8+^ has eight terminal OH groups that represents opportunity for exchange with a ligand *via* solvent‐assisted linker incorporation (SALI).^[71]^ Hu *et al*. have introduced 4‐(2,6‐Dimethylpyridin‐4‐yl)benzoic acid as a pyridyl Lewis base and B(2,6‐C_6_F_2_H_3_)_3_ as the Lewis acid and selectively reduced nitroolefins to nitroalkanes.^[72]^ It showed excellent recyclability and functional group tolerance, efficiently reducing a variety of groups without any decrease in product yields.

**Figure 3:**
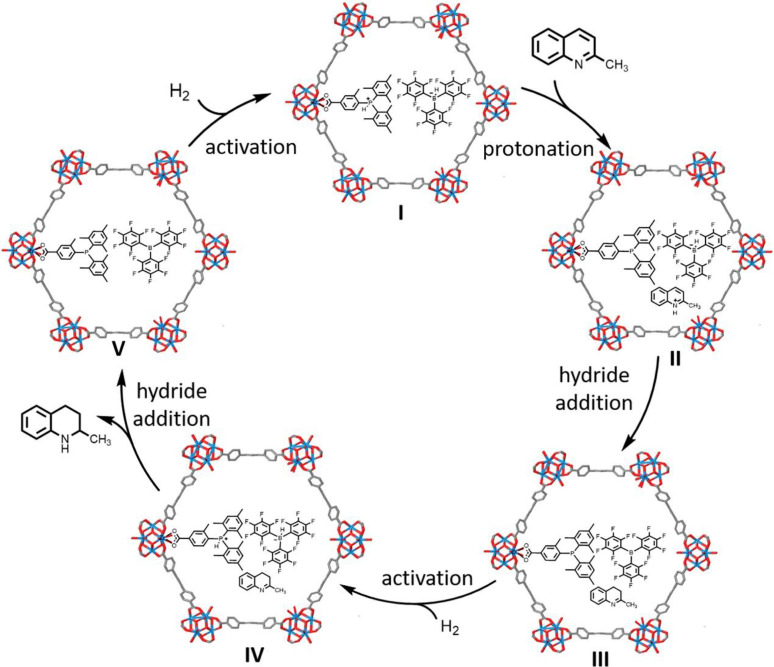
A MOF pore (NU‐1000) with a triarylphosphine anchored to one Zr node in the pore. Incorporation of guest BCF forms a FLP for the reduction of a quinoline *via* a suggested mechanism. Adapted with permission from Ref. [73]. Copyright 2023 American Chemical Society.

Ma's group observed poor performance with their MIL‐101(Cr)/DABCO/B(C_6_F_5_)_2_(Mes) system towards reduction of nitrogen heterocycles.^[41]^ However, switching to NU‐1000 and anchoring (2,4,6‐Me_3_C_6_H_2_)_2_P(2‐Me‐4‐CO_2_HC_6_H_3_) through the SALI approach gave a P‐based Lewis base and excellent results, with the reduction proceeding through a standard FLP type mechanism (Figure 3).^[73]^

It is relatively challenging to synthesise a MOF with an inherently free P group, due to the likelihood of coordination to the metal node and possible oxidation under MOF crystallisation conditions.[^74^, ^75^, ^76^, ^77^] However, the covalent post‐synthetic modification strategy allows for a relatively straightforward addition of phosphines. In NH_2_‐MIL‐101(Cr), the terephthalic acid linker can be exchanged for 2‐amino terephthalic acid, which has a primary amine group pointing towards the pore. In a broader design strategy sense, this is an easily accessible point for which to graft additional functionality through simple nucleophilic substitution or condensation reactions. Xu *et al*.^[78]^ demonstrated this strategy through attaching a triarylphosphine group into the pore site *via* a condensation reaction with 2‐(diphenylphosphino)benzaldehyde and the pendant amine of the linker. This FLP‐MOF continued the trend in selectivity for imine reduction in α,β‐unsaturated imines, though with PhMe_2_SiH as the reducing agent.^[78]^

Given the vast literature on post‐synthetic modification of MOFs through modifying the metal node or the organic linker, it is perhaps surprising that there are not more FLP‐MOFs that have taken advantage of this approach. Post‐synthetic modification is a straight forward design strategy that offers a substantial amount of variety and control for the FLP functionality.

**Figure 4:**
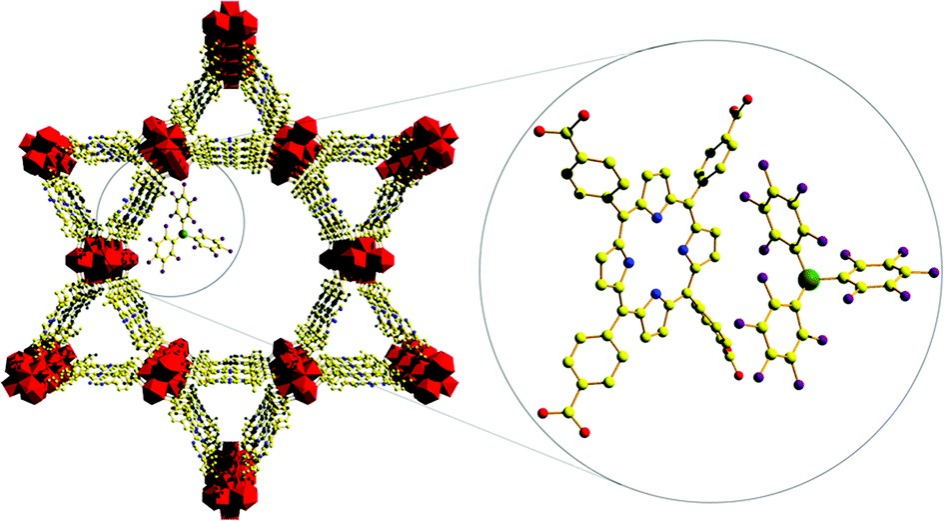
Use of the Lewis basic MOF‐545 backbone with a guest BCF molecule. Reproduced from Ref. [79] with permission from the Royal Society of Chemistry.

An alternative approach to the incorporation of FLPs within MOFs is to use the organic linker as either the Lewis base or acid, and then add in the opposing component as a guest within the pore structure. Dyson's group have reported MOF‐545 (molecular formula=Zr_6_O_8_(H_2_O)_8_(TCPP‐H_2_)_2_, TCPP=tetrakis(4‐carboxyphenyl)porphyrin), where the porphyrin moiety contained the N Lewis base and BCF was added as the Lewis acid (Figure 4). This system enabled the reduction of CO_2_ to a methoxyborate, but also in the loss of BCF.^[79]^ The same group have also demonstrated the analogous use of a MOF with a boron based linker instead. SION‐105 (molecular formula=Eu(tctb)(H_2_O), tctb=tris(*p*‐carboxy)tridurylborane), where the key motif to the organic linker is a triarylborane. SION‐105 proved to be quite active in the reaction of aryl *o*‐diamines with CO_2_ and Ph_2_SiH_2_ as a reducing agent to form the corresponding fused benzimidazoles^[80]^ – a reaction that has been shown to proceed in an FLP mediated way.^[81]^

A major focus of the MOF field has been around CO_2_ capture, for which Lewis basic sites with MOF pores/channels was a key target.[^82^, ^83^] Thus, there are a substantial amount of known Lewis basic MOFs that have potential as hosts for a Lewis acid guest and so form a FLP‐MOF.

**Figure 5:**
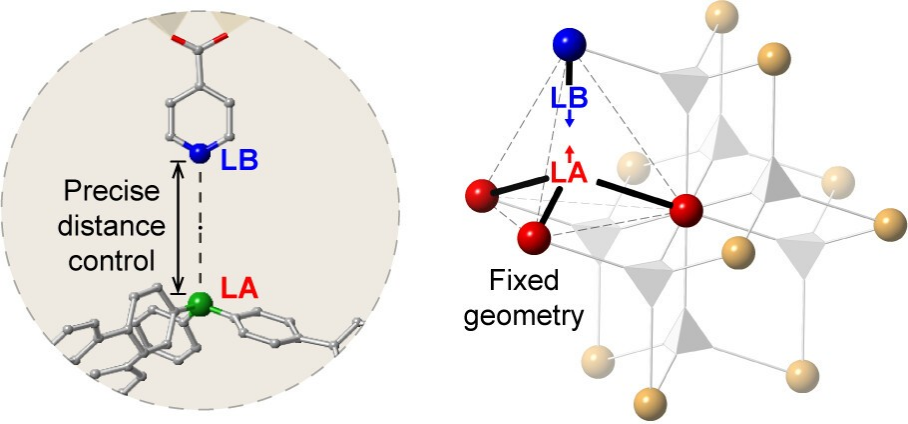
Switching out a tetratopic linker for a Lewis acidic tritopic and Lewis basic monotopic linker in PCN‐521 to create fixed geometry FLP sites within MOF pores. Adapted with permission from ref. [96] Copyright 2024 American Chemical Society.

There are relatively few MOF/FLP systems that have combined both the Lewis acid and base intrinsically in the structure.[^84^, ^85^] This is despite the plethora of reports of Lewis acid catalysed reactions in MOF systems and the ability to control the Lewis acidity of the metal node,[^86^, ^87^, ^88^, ^89^, ^90^, ^91^, ^92^, ^93^, ^94^] which suggests this area is ripe for exploration in the future. One extremely interesting approach to this that taken by Chen *et al*., who used the “molecular vice” approach^[95]^ to systematically control the distance between N and B moieties (Figure 5).^[96]^ In this approach, Zhou's fluorite MOF PCN‐521 (molecular formula=[Zr_6_(μ_3_‐OH)_8_(OH)_8_)]MTBC_2_, MTBC=4′,4′′,4′′′,4′′′′‐methanetetrayltetrabiphenyl‐4‐carboxylate)^[97]^ has part of the tetratopic linker (MTBC) replaced by a combination of monotopic base (e. g. isonicotinic acid) and a tritopic linker (tris(2′,3′,5′,6′‐tetramethylbiphenyl‐4‐carboxyphenyl)borane), leading to the N and the B centres pointing at each other. This approach allows for controlling the length of the N−B interaction through choice of the base.^[96]^

**Figure 6:**
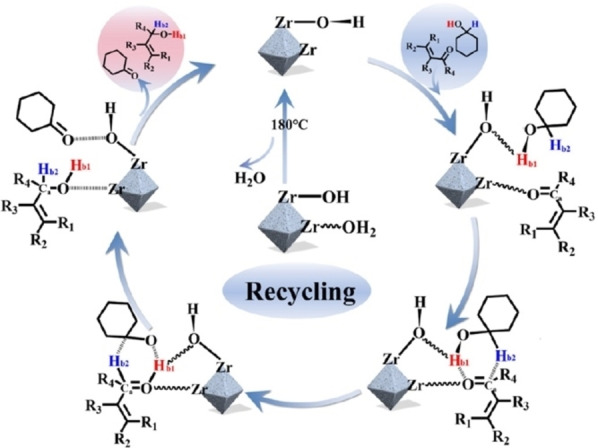
Generation of a Lewis acidic, coordinatively unsaturated Zr, with a neighbouring Lewis basic hydroxy group for FLP formation on a MOF metal node. Adapted with permission from Ref. [99]. Copyright 2024 American Chemical Society.

A coordination defect‐induced strategy has been employed by Liang *et al*.^[98]^ and Zhong *et al*.[^99^, ^100^] to generate Lewis acidic metal sites with the oxygen from the metal node acting as a neighbouring Lewis basic site (Figure 6) – both systems demonstrated good FLP activity. This concept of utilising neighbouring sites in the metal cluster seems open to significant exploration as it takes advantage of a precise distance between the reactive sites and the number of defects within the MOF can be readily controlled.

Finally, Das *et al*. have generated some design rules for the structural relationship between B and N ligands in a MOF pore which offer an interesting guide for experimentalists.^[101]^

**Figure 7:**
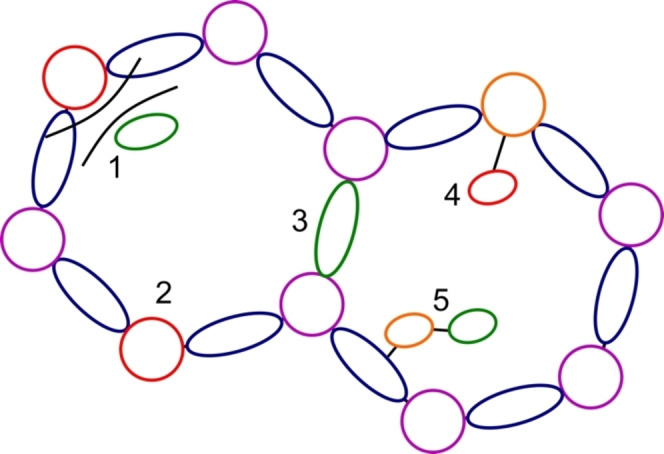
Design rules for FLP‐MOFs as outlined in the main text. Purple=metal node, blue=organic linker, red/green=Lewis acid/basic functionality, orange=functionalisable group.

Overall, the structural rigidity, defined pore size and ability to act as heterogeneous scaffolds for homogeneous style catalysis means that MOF/FLP systems are a very attractive area for future exploitation. The covalent/coordination modification of known MOFs that have accessible sites and a large pore size is a simple design approach that offers substantial scope for catalytic reactions. This can be based on a “neutral” MOF, but there are a large number of reported systems that are already Lewis acidic/basic. It is challenging to rationally design a MOF with FLP functionality as part of the core structure, but the molecular vice approach is a straightforward and scalable system that offers opportunity. A set of guidelines for designing a FLP‐MOF catalyst are summarised in Figure 7. The MOF needs a large enough pore volume to incorporate the small molecules and possibly a guest Lewis acid/base (1). The FLP functionality can be part of the MOF itself, but it is easier to have an active metal node (2) or organic linker (3). Post‐synthetic modification of the metal (4) through desolvation/linker exchange, or the organic linkers (5) is a straightforward way of introducing functionality.

### 2.2 Covalent‐Organic Frameworks

Covalent‐organic frameworks (COFs) are another class of attractive material with similar properties to MOFs, but are instead made up of covalently bonded light elements.^[102]^ Their large pore sizes and volumes, high stability and designable functionalities make them excellent candidates for heterogeneous catalysis.[^103^, ^104^, ^105^, ^106^, ^107^, ^108^] There are three main strategies to prepare FLP‐COFs: (*i*) combining a Lewis acidic/basic functional group in the organic backbone with the corresponding guest, (*ii*) post‐synthetic modification to covalently bind the Lewis/acid base with the reverse as a guest and (*iii*) direct incorporation of both parts of the the FLP in the organic framework. In a manner comparable to that of the FLP‐MOFs, the incorporation of catalysts is often performed *via* post‐synthetic modification, as the stability of the catalytic functional groups is often incompatible with the COF synthetic conditions. However, thanks to the characteristic strength of the covalent linkages and the ready availability of pendant reactive linkages on the COF backbone, it is relatively straightforward to graft on an active catalyst.[^109^, ^110^, ^111^, ^112^]

**Figure 8:**
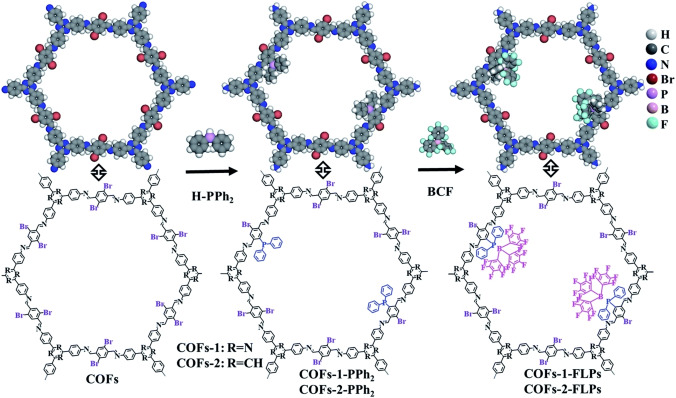
Illustration of a post‐synthetic modification strategy to graft a phosphine group into a COF. Reproduced from Ref. [113] with permission from the Royal Society of Chemistry.

Given the versatility of COFs in this space, there are remarkably few examples of COF/FLP systems. The first example was reported in 2022 by Liu *et al*., who took a COF with a linker featuring an un‐masked aryl‐bromide motif^[113]^ and performed a simple Ullmann‐type coupling with diphenylphosphine (Figure 8). The addition of BCF as a guest gave rise to a COF/FLP system that selectively hydrogenated alkynes to Z‐alkenes with good conversion and excellent recycling performance.^[113]^

**Figure 9:**
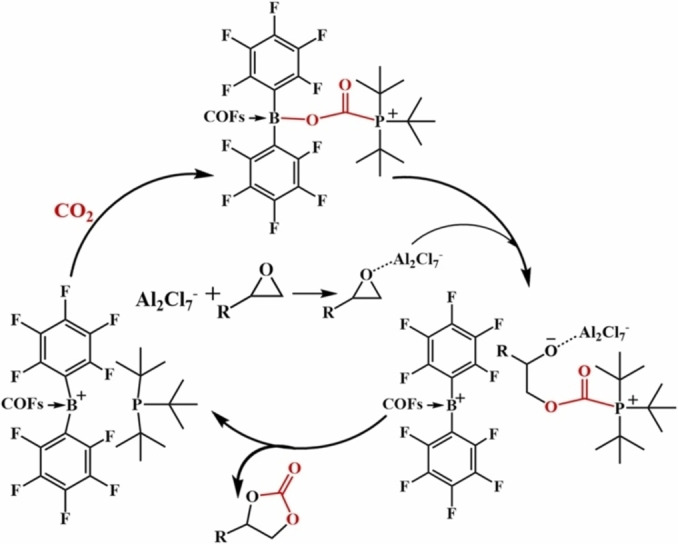
The possible mechanism for the activation of CO_2_ by a FLP‐COF for insertion into epoxides. Figure reproduced from Ref. [115], copyright 2024 with permission from Elsevier.

The same group have used the imine backbone of a COF to graft on Lewis acidic functionality through ‐B(C_6_F_5_)_2_ motifs. They performed a hydroboration to add HB(C_6_F_5_)_2_ across the imine, which in combination with a guest 1,8‐diazabicycloundecano‐7‐ene (DBU) gave rise to a COF/FLP system. This system was able to reduce terminal alkynes to alkenes with good conversion and selectivity, where BCF and DBU without the COF gave no conversion.^[114]^ An alternative approach datively coordinated ClB(C_6_F_5_)_2_ to an imine in the COF, followed by chloride abstraction with AlCl_3_ to generate a borenium ion. Using ^t^Bu_3_P as the Lewis base gave a catalyst that could insert CO_2_ into epoxides to form cyclic carbonates. The suggested mechanism for activation is comparable to standard FLP activation of CO_2_. (Figure 9).^[115]^

In a similar method to the chiral FLPs bound to MOF pore sites,[^69^, ^70^] Ma's group have grafted chiral amines onto a COF backbone.^[116]^ In combination with BCF and a high pressure of H_2_ (60 bar), this is an active catalyst for asymmetric olefin hydrogenation with good yields and reasonable enantiomeric excess.

Zhong *et al*. took a completely different approach and intrinsically embedded the FLP functionality in their system from the initial synthesis. To do this, a Schiff base derived network obtained from condensation of melamine and terephthaldehyde^[117]^ was partially substituted with 4‐formylphenylboronic acid.^[118]^ This resulted in the formation of B/N pairs in the carbon scaffold, which selectively reduced α,β‐unsaturated ketones to the corresponding unsaturated alcohol.

## 3 Polymers

Polymers featuring FLP structures within them have exploded in prominence since around 2017. Embedding FLP functionality within polymeric structures offers a number of opportunities ranging from heterogeneous catalyst immobilisation to imbuing smart materials with “self‐healing” capabilities. There are three ways of creating the FLP reactive centres in polymeric structures: (*i*) Lewis acid/base functionalised polymers with guest partner base/acid, (*ii*) polymer blends of Lewis acid/base functionalised polymers and (*iii*) bifunctional FLP polymers.

Additionally, supramolecular polymers are a type of polymer based on monomeric units being held together by directional and reversible non‐covalent interactions.[^119^, ^120^] In this case there are several reported stacks of FLPs that result in the generation of supramolecular polymers.

### 3.1 Lewis Acid/Base Functionalised Polymers

The simplest way of incorporating FLP functionality into a polymeric structure is to take a Lewis acidic or Lewis basic polymer[^121^, ^122^] and add the corresponding pair as a monomeric species. Perhaps surprisingly, this space is relatively under explored given this simplicity – resulting in plenty of opportunity for future discovery.

The first polymeric supported FLP was reported by Thomas *et al*. in 2017.^[123]^ They prepared a phosphine based polymer *via* Yamamoto coupling of tris(*p*‐bromophenyl)phosphine derivatives and generated a heterogeneous FLP with the addition of BCF. This catalyst activated hydrogen, which was demonstrated with H/D isotope scrambling experiments, and also proved superior to its homogeneous congener for the hydrogenation of diethyl benzylidenemalonate.^[123]^

In 2018 Willms *et al*. followed this with a polyamine and BCF analogue that was active towards the hydrogenation of alkenes.^[124]^

### 3.2 Polymer Blends

**Figure 10:**
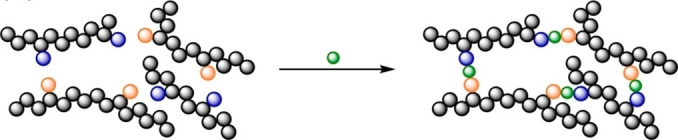
Cross‐linking of polymeric FLP blends (blue=Lewis base, orange=Lewis acid) with small molecules such as DEAD or CO_2_ (green). Adapted with permission from ref. [125] Copyright 2017 American Chemical Society.

There are a considerable number of polymeric blends – *i. e*. combinations of a Lewis basic polymer and a Lewis acidic polymer, which have demonstrated increasingly sophisticated reactivity. The Shaver group have prepared a range of block co‐polymers based on the polymerization of styrene and 4‐styryl‐diarylphosphine or 4‐styryl‐diarylborane. These polymeric FLP blends can be reversibly cross‐linked through FLP activation of diethyl azodicarboxylate (DEAD),[^125^, ^126^] CO_2_ ^[127]^ or through ring‐opening epoxides to make self‐healing gels (Figure 10).[^128^, ^129^] The ring‐opened epoxide cross‐linked polymers can further react with CO_2_ to form cyclic carbonates and reform the FLP polymeric blend.^[130]^ Similar behaviour has also been reported by Yan's group^[131]^ and very recently by Holland *et al*. in a polyamine/polyborane blend for the first time.^[39]^

**Figure 11:**
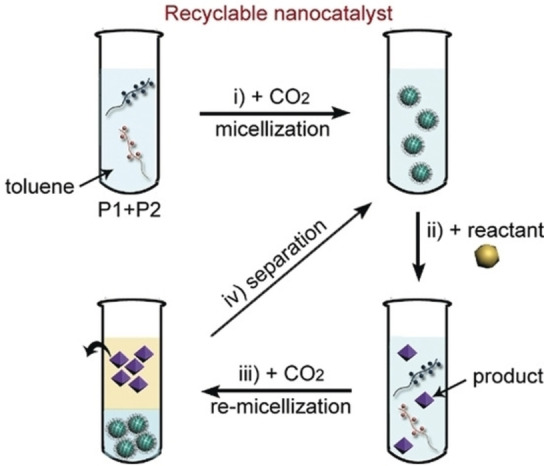
FLP‐polymer blends as recyclable nanocatalysts: i) FLP‐polymer micellization triggered by CO_2_; ii) Cross‐linked polymers catalyse the reaction between a reactant and CO_2_; iii) additional CO_2_ induces re‐micellization of FLP‐polymer; iv) separation of products and re‐dispertion of the FLP‐polymer nanoparticles for a new catalytic cycle. Reproduced from Ref. [131] with permission from Wiley.

The FLP‐polymer blends in these studies act as recyclable nanocatalysts for the utilization of CO_2_, resulting in a generalised design strategy that can be built upon. When the polymer blends are exposed to CO_2_ the FLP groups activate the CO_2_, forming cross‐links between the polymers and generating micelles. Adding a reactant to form a new product, such as a carbonate, breaks the cross‐links resulting in a disruption of the micelle. Further addition of CO_2_ regenerates the micelles, allowing for separation of the nanocatalyst from the product (Figure 11). This is an effective and intriguing strategy for making a recyclable FLP catalyst.

### 3.3 Bifunctional Polymers

**Figure 12:**
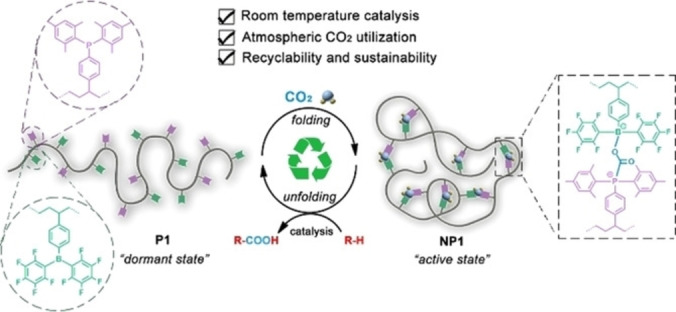
Cross‐linking a B/P block co‐polymer through FLP activation of CO_2_. Copyright 2019 Wiley. Reproduced from Ref. [132] with permission from Wiley.

For polymers that contain the FLP within one polymer chain there are several options. In the first instance, Yan's group have reported a single block co‐polymer with blocks derived from styrene (as a spacer), 4‐styryl‐di(pentafluorophenyl)borane and 4‐styryl‐dimesitylphosphine.[^132^, ^133^] These polymers adopt a relative relaxed state by themselves, but when CO_2_ is introduced they coil up into an active state. The FLP activates the CO_2_ to cause cross‐links, and the CO_2_ can act as a C1 source for the formation of carboxylic acids (Figure 12) or cyclic carbamates.[^132^, ^133^]

Very recently Latif *et al*. have prepared a polymer which they tentatively assign to contain an intramolecular FLP motif.^[134]^ Under typical polymerization conditions it is very hard to ensure optimal vicinal positioning of a Lewis acidic and basic group. In this example they designed a norbornadiene‐derived FLP adduct as a caged monomer for vinyl addition polymerization with pentylnorbornene as the copolymer. The FLP was then unmasked through a photoactivation step to leave an “intramolecular” dimestiylphosphine/bis(pentafluorophosphine) borane group held apart by a short carbon backbone.^[134]^

### 3.4 Supramolecular Polymers

In 2020 Meijer reported the first FLP supramolecular polymer,^[120]^ which was formed by the co‐polymerisation of O‐bridged triphenylborane and triphenylamine.^[135]^ The two co‐monomers form blocks within the polymeric structure, with an FLP‐type interaction between each block. Interestingly, this polymer displays circularly polarised exciplex emission as a result of the B−N FLP interactions. Alternating borane/amine monomer units within the polymeric stack have not yet been realised,^[136]^ but there are very exciting opportunities for optoelectronic applications in this space.

## 4 Macrocycles and Cages

Macrocycles and cages have received vast amounts of attention from supramolecular chemists over the past few decades. They have tremendous potential in applications as diverse as molecular machines,[^137^, ^138^, ^139^] enzyme mimics,[^140^, ^141^, ^142^] sensing,^[143]^ drug delivery,^[144]^ catalysis[^145^, ^146^, ^147^, ^148^] and optoelectronics.[^149^, ^150^] These two systems draw broad comparisons to the active sites of enzymes, thanks to the confined cavity that they create within. The well‐defined and structurally controlled nature of the cavity lends itself to be a platform for FLP mediated catalysis.

Careful design of the macrocycle/cage can create an active site that is sufficiently hydrophobic as to increase the local concentration of reagents, can stabilize transition states, can improve reaction rates and can direct enantioselective outcomes.[^151^, ^152^, ^153^] Rebek's rule for the volume of the guest that can bind in a supramolecular host is that the guest occupies approximately 55 % of the volume of the host.^[154]^ This makes it quite limiting if the host contains only one part of the Lewis pair, as the host would have to find room for the opposite congener and the precursors in any reaction. Therefore, it is reasonable to suggest that the majority of active catalysts in this space will have both Lewis acidic and basic motifs in the host structure.

The inclusion of multiple Lewis acid/base groups in the backbone of the host offers the opportunity for more complex reactivity involving the transfer of more than two electrons, or cooperative selection between substrates at multiple active sites.[^40^, ^155^]

### 4.1 Macrocycles

**Figure 13:**
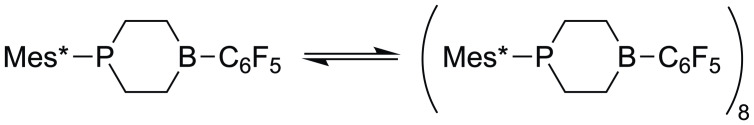
Aggregation of a six‐membered cyclic P/B FLP into a cyclooctameric macrocyclic ring.^[163]^

There are numerous reports on the use of FLPs to generate macrocycles,[^150^, ^155^, ^156^, ^157^, ^158^, ^159^, ^160^, ^161^] but relatively few true FLP macrocycles. In 2012, Jäkle's group reported the first B/N macrocycle that had no dative B−N interactions,^[162]^ and which could be considered to be a FLP macrocycle. In 2019 Erker reported the aggregation of a six‐membered cyclic P/B FLP into a cyclooctameric macrocyclic ring (Figure 13).^[163]^ This cyclooctamer is the thermodynamically favoured product at low temperature and in crystalline form, but in solution it disaggregates back to the monomeric form and thus no additional reactivity from the macrocyclic system has been observed.^[163]^

**Figure 14:**
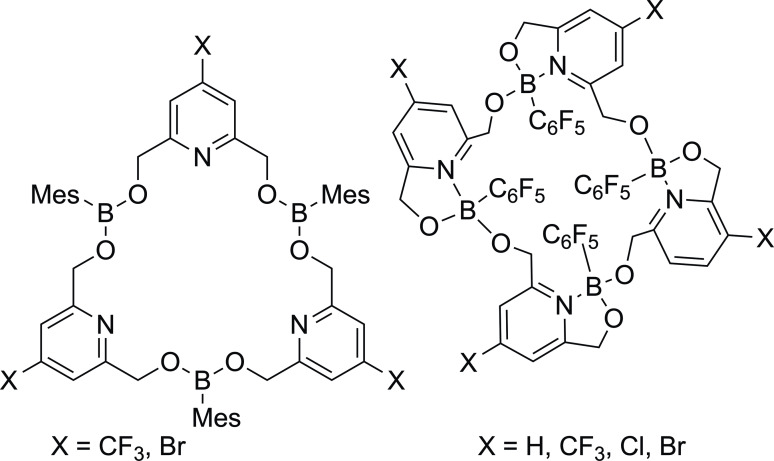
Two examples of FLP macrocycles, featuring no B−N linkage when prepared with MesBH_2_ (left) and dative B−N bonds when synthesized with (C_6_F_5_)BH_2_ (right).^[40]^

The closest example to a macrocyclic FLP catalyst was reported by Stephan in 2022 from the reactions of the primary boranes (C_6_F_5_)BH_2_ and MesBH_2_ with 2,6‐pyridinedimethanol. The use of (C_6_F_5_)BH_2_ resulted in a macrocycle with a dative B−N bond, but the bulkier mesityl group appears to suppress this (Figure 14). The addition of BCF simply results in coordination of this to the pyridinic nitrogen, with no further small molecule activation.^[40]^ This suggests the opportunity for macrocyclic FLPs exist, but there are significant synthetic challenges.

An alternative class of system was reported by Stephan in 2012,^[164]^ which took a sterically unencumbered N‐benzylamine and converted it to a [2]rotaxane with 24‐crown‐6 and 22‐crown‐6 as the wheels. The N‐benzylamine formed a classical Lewis pair with BCF, but the [2]rotaxane formed a FLP that could successfully activate H_2_.

### 4.2 Cages

In much the same way as the well‐defined pores of the MOFs/COFs discussed earlier, supramolecular cages offer the possibility of controllable reactivity centres. However, perhaps for similar reasons to the macrocycles and the synthetic challenges associated with endohedrally functionalizing cavities,^[165]^ there are limited examples of supramolecular cage FLPs in the literature.

**Figure 15:**
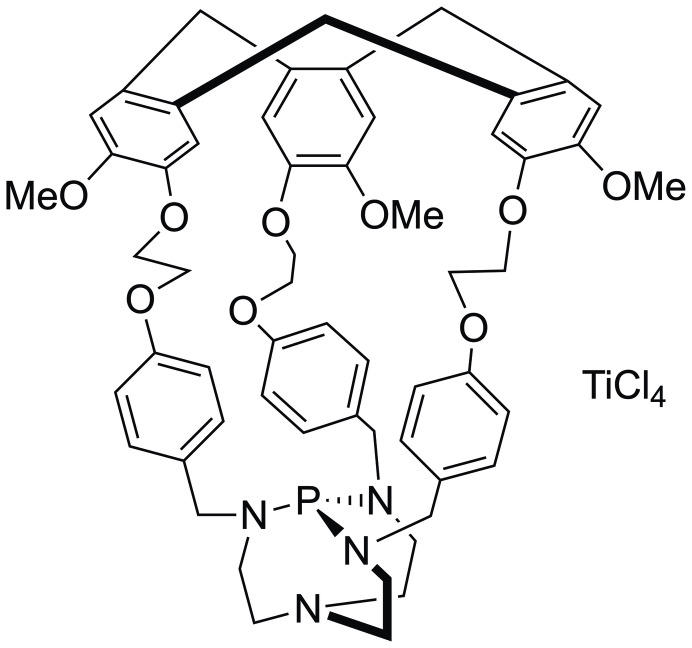
An Verkade's superbase endohedrally functionalized hemicryptophane with TiCl_4_ as a corresponding Lewis acid is an active catalyst for the Morita–Baylis–Hillman reaction.^[166]^

One example from the group of Martinez encapsulated Verkade's superbase within a hemicryptophane host (Figure 15).^[166]^ On its own, this cage demonstrated little reactivity, but with TiCl_4_ as a Lewis acid, provided a reasonable catalyst for the Morita–Baylis–Hillman reaction. The confinement of Verkade's superbase within the host prevents the reaction of the base with the TiCl_4_. The group investigated how the linkage length, and thus cage size and shape, influences the reactivity. Increasing the linkage creates a larger pore size, which led to an increase in activity, demonstrating the importance of cavity size on the reactivity.^[167]^

## 5 Summary and Outlook

Frustrated Lewis pairs offer outstanding capabilities for metal‐free catalysis. However, the current generation of FLPs are typically used in homogeneous systems and degrade when exposed to oxygen and water. Immobilising at least one part of an FLP within a supramolecular assembly can result in improved reactivity, and/or different chemo‐/regio‐/enantioselectivity, thanks to the ability to control the local environment around the FLP.

Supramolecular assemblies offer a customisable route to spatial control of molecular components. These materials can be readily prepared from convenient precursors and offer enormous potential for chemical modification and optimisation. Supramolecular FLPs are the perfect solution to the current challenges of FLP catalysis: molecular geometry is strictly enforced, and for MOFs/COFs and polymers particle engineering, monolith growth or surface immobilisation provides easy access to heterogeneous reaction conditions. Furthermore, interference from physisorbed oxygen is expected to be reduced in any microporous FLPs lacking redox‐active open metal sites, due to its low polarizability and quadrupole moment disfavouring adsorption compared to dinitrogen, solvent molecules and reactant species

There has been a remarkable growth in this field in the past 7 or 8 years, but there remain significant opportunities for future endeavours. In particular, FLP‐MOFs/FLP‐COFs offer the opportunity for supported‐but‐homogeneous‐in‐function catalysts, which is beneficial for high throughput and catalyst recovery. The pioneering work of groups in these areas have offered a number of design strategies, of which post‐synthetic modification within a pore site is the most straight forward to make the most rapid progress in. FLP‐polymers have demonstrated excellent activation of small molecules such as CO_2_ to generate cross‐linked blends, and this is a space that is seeing current and rapid progress.

In our opinion the true benefit of utilising supramolecular FLPs will be realised in three instances:

- where the recyclability benefits of heterogeneous catalysts can be combined with the reactivity benefits of a homogeneous mode of operation
- where the well‐defined environment imparts inherent reactivity control unavailable with monomeric analogues – i. e. enantioselectivity
- where multiple FLP units within one pore site/cavity can cooperate to increase selectivity or mediate multi‐electron processes.

These all pose significant synthetic challenges, and are perhaps most likely to be achieved by FLP‐MOFs, FLP‐COFs and FLP‐polymers. Despite these challenges, as we have shown in this review, the field is making significant strides to realise a bright future of immobilised, noble metal free catalysts.

## Conflict of Interests

The authors declare no conflict of interest.

## Biographical Information

*Paige Hawkins obtained her Bachelor's degree in Chemistry from the University of Wolverhampton in 2023. At present, she is pursuing her Ph.D. in heterogeneous catalysis at Keele University under the supervision of Dr Chris Hawes and Dr Peter Matthews. Her main research focus is on confining frustrated Lewis pairs within metal–organic frameworks, by designing and synthesising novel MOF/FLP systems*.

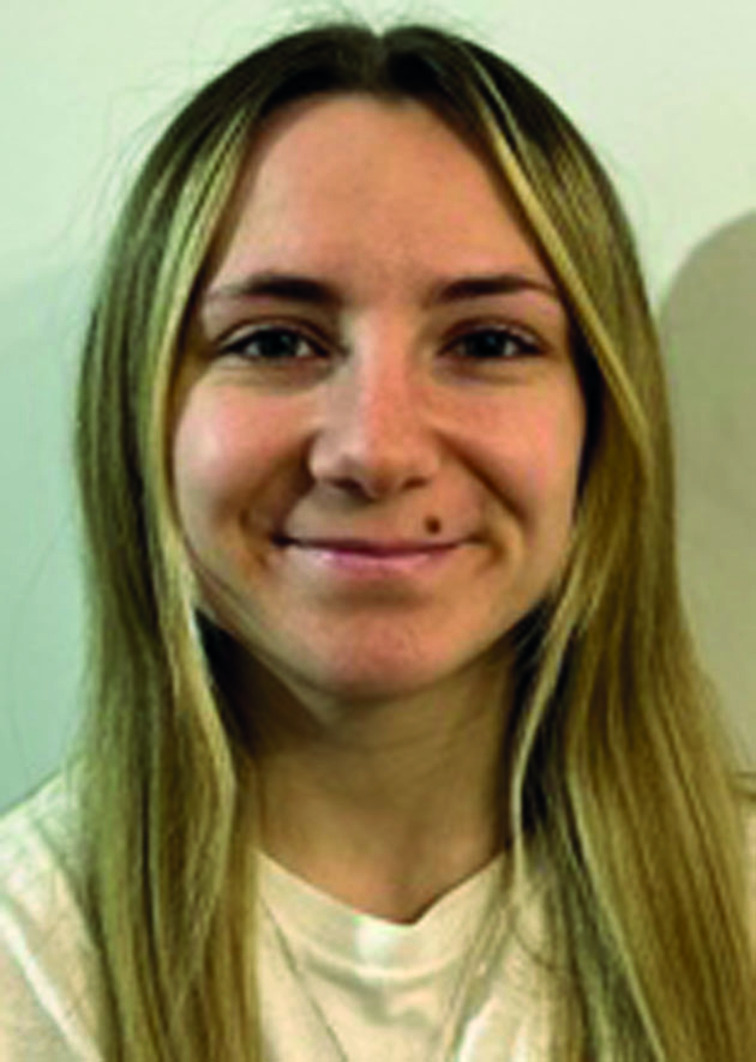

## Biographical Information

*Chris Hawes completed his Ph.D at the University of Canterbury, New Zealand in 2012, and following postdoctoral positions at Monash University and Trinity College Dublin, moved to Keele University as a Lecturer in Inorganic Chemistry in 2017. His research interests are focused on structural chemistry and ligand design in functional metallo‐supramolecular systems and MOFs*.

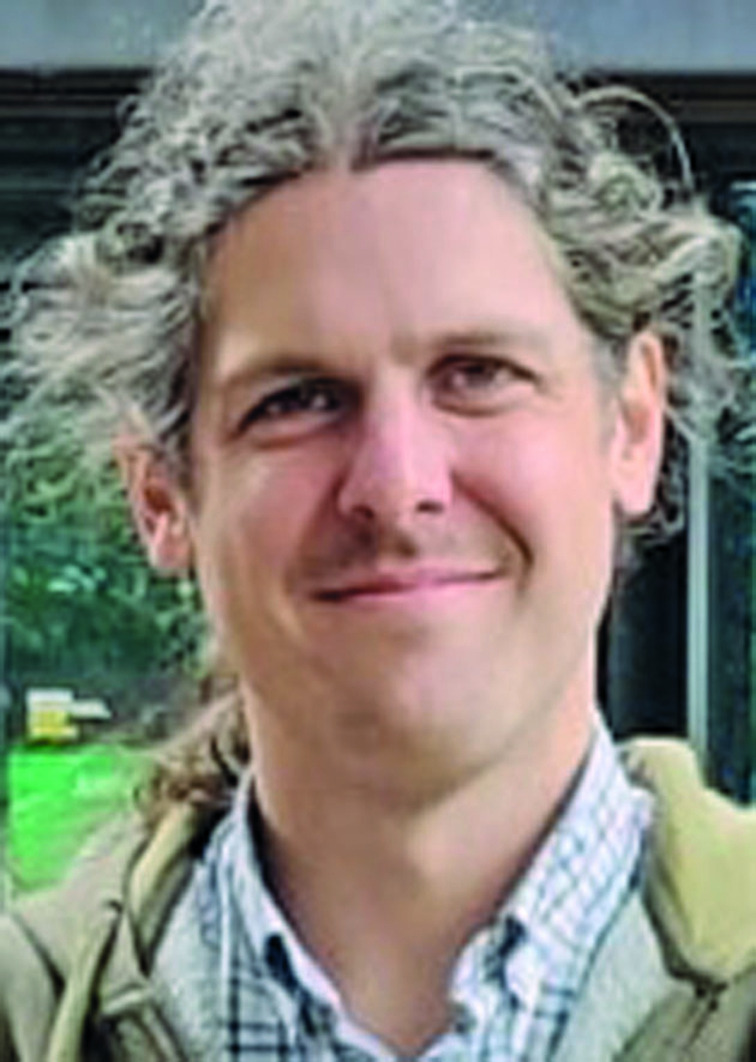

## Biographical Information

*Peter Matthews studied for his Ph.D at the University of Cambridge (2012–16) before undertaking a postdoctoral position at the University of Manchester. He started at Keele in 2018 as a Research Fellow and was appointed Senior Lecturer in Inorganic and Materials Chemistry in 2023. He is interested in the interface between main group molecules and bulk materials*.